# Comparison of polyurethane foam dressing and hydrocolloid dressing in patients with pressure ulcers: A randomized controlled trial protocol

**DOI:** 10.1097/MD.0000000000024165

**Published:** 2021-01-15

**Authors:** Yan Jin, Jun Li, Shuai Wu, Fei Zhou

**Affiliations:** a Diagnosis and Treatment Center for Chronic Wounds, Hangzhou Geriatric Hospital; b Department of Surgery, Community Health Service Center, Yuhang District; c Diagnosis and Treatment Center for Chronic Wounds, Hangzhou Geriatric Hospital, Hangzhou, Zhejiang, China.

**Keywords:** hydrocolloid dressing, polyurethane foam dressing, pressure ulcer, protocol

## Abstract

**Background:**

We conduct this randomized controlled trial protocol for the comparison of the influence of the hydrocolloid dressing and polyurethane foam dressing in the treatment of pressure ulcers (PUs) patients.

**Methods:**

This study will be implemented from February 2021 to February 2022 at Hangzhou Geriatric Hospital. The experiment was granted through the Research Ethics Committee of Hangzhou Geriatric Hospital (C5259033). Criteria for inclusion: patients older than 18 years of age who have been diagnosed with PU. If the patient suffers from more than a PU, only the ulcer with largest diameter is evaluated.

Criteria for exclusion:

(1) hypersensitivity or allergy to the substances in dressings;
(2) patients with diabetic foot or venous ulcers; and
(3) serious disease.

The major result is rate of PU healing or ulcer epithelialization tissue. The secondary result is the changes in the area of ulcer in cm and cost-effectiveness. The analysis of all the data are conducted with the software of IBM SPSS Statistics for Windows, version 20 (IBM Corp., Armonk, NY).

**Results:**

Table 1 will show the comparison of clinical outcomes between 2 groups.

**Conclusion:**

This study can develop an evidence-based protocol to identify optimal dressings for patients with PUs.

**Trial registration number:**

researchregistry6294

## 1 Introduction

Pressure ulceration is an important problem of healthcare in the world, placing a great burden on the resources of healthcare.^[1,2]^ Pressure ulcers (PUs) are usually hard to cure and can cause a serious negative impact on the life quality and mortality of patients.^[3,4]^ The generation of PU is greatly affected via a variety of risk factors, involving various conditions that result in malnutrition, the lack of sensation, and hypermobility.^[5,6]^ In recent years, the epidemiological data related to PUs in the United States has been relatively limited, with the annual incidence estimated at between 1 million and 3 million.^[7]^ Among all the inpatients, the reported prevalence varies significantly, with a total of 5% to 15% of patients affected, but patients’ proportion affecting in ICU is still high.^[8]^ Despite PUs are generally caused by the poor condition of health and some other diseases, in many cases it can be avoided. Thus, our target is to prevent the PU, which is all more important in view of the high treatment cost and challenges. For the effective prevention approaches, its fundamental elements involve proper nutrition, the use of appropriate support surfaces, frequent repositioning, and moisture management.^[9–11]^

Polyurethane foam dressings have been developed for many years and have been utilized to treat the wounds with mild to moderate exudate.^[12]^ It is easy to utilize and then remove and is beneficial to the process of healing. Recent studies have reported that polyurethane foam dressings were effective in treating PUs. Other articles also recommended to prevent PUs by using a hydrocolloid dressing, including ulcers caused by noninvasive ventilation.^[13]^ So far, the clinical trials and systematic reviews have not provided sufficient evidence to demonstrate their effectiveness. Therefore, we conduct this randomized controlled trial protocol for the comparison of the influence of the hydrocolloid dressing and polyurethane foam dressing in the treatment of PUs patients.

## 2 Methods

This study will be implemented from February 2021 to February 2022 at Hangzhou Geriatric Hospital. The experiment was granted through the Research Ethics Committee of Hangzhou Geriatric Hospital (C5259033) and recorded in research registry (researchregistry6294). Sequence of random numbers is generated by a computer. Sequentially numbered sealed opaque envelopes are used for the concealment of random numbers. All the patients taking part in our experiment are randomly divided to polyurethane foam dressing or hydrocolloid dressing group, and each group includes 48 participants.

### 2.1 Inclusion and exclusion criteria

Criteria for inclusion: patients older than 18 years of age who have been diagnosed with PU. If the patient suffers from more than a PU, only the ulcer with largest diameter is evaluated. Criteria for exclusion:

(1) hypersensitivity or allergy to the substances in dressings;
(2) patients with diabetic foot or venous ulcers; and
(3) serious disease.

### 2.2 Intervention

The standardized preventive intervention will be given to all patients for the reduction of pressure and experience a process of PU healing treatment.

The process of healing cure involves a total of 3 aspects:

(1) Every time the research dressing is changed, wound needs to be washed. The skin cleaners and local antiseptics should be avoided because they are cytotoxic to new granulating tissue.
(2) The process of drying must also be delicate. The rough materials may cause minor wounds in wound bed, thereby interfering with the process of healing and enhancing the infection risk. The wound bed should be moist and the edges of the wound should be clean and dry. Care should be taken to avoid damaging healthy tissue during cleansing and drying procedures.
(3) The prevention of bacterial infection should be strengthened. The aseptic techniques should be utilized wherever possible, involving utilizing the clean gloves. Proper debridement and healing procedures can reduce the infection risk. If the patient suffers from more than a PU, the most contaminated ulcer should be left for treatment at the end.

Polyurethane foams: These dressings have the hydrophilic structure and are derived from polyurethane. They have a high exudates absorption and a high autolysis debridement capacity, and can prevent the wound bed from drying, leaving no decomposition, and residuals. Furthermore, they avoid odors, stains and leakages, keep the skin around the wound intact, and do not cause traumas during removal. These dressings are suitable at all the PU stages. When PU infections occur, the frequent monitoring and modification may be necessary.

Hydrocolloids: These dressings are composed of carboxymethyl cellulose and some other water active compounds, adhesion substances, or hydrocolloids to offer the absorption capacity. These dressings are covered with a layer of polyurethane to make them semi occlusive or occlusive properties. Furthermore, they absorb exudates and necrotic residuals by forming a gel with special color and odor characteristics, creating a slightly acid environment with bacteriostatic properties. In addition, they decrease the friction. These dressings are suitable for uninfected PU.

### 2.3 Outcome

The major result is rate of PU healing or ulcer epithelialization tissue. The secondary result is the changes in the area of ulcer in cm and cost-effectiveness.

### 2.4 Statistical analysis

The analysis of all the data are conducted with the software of IBM SPSS Statistics for Windows, version 20 (IBM Corp., Armonk, NY). The data obtained are represented through the proper features, for example,. standard deviation, and mean, median as well as percentage. And independent *t* tests and χ2-tests are respectively utilized to analyze the categorical variable and continuous variable. When *P* is less than .05, the efficacy is viewed to be statistically significant.

## 3 Result

Table 1 will show the comparison of clinical outcomes between 2 groups.

**Table 1:** Comparison of clinical outcomes between 2 groups.

	Polyurethane foam dressing group (n = 48)	Hydrocolloid dressing group (n = 48)	*P* value
Rate of pressure ulcer healing			
Changes in the area of ulcer			
Medical cost			
Frequency of dressing			
Length of hospitalization			
Complications			

## 4 Discussion

PUs (also known as bed sores, decubitus ulcers, and pressure sores) refers to the wounds to the underlying tissue and skin resulted from rubbing or pressure.^[14,15]^ They generally generate in the bony parts of the body or in places that bear pressure or weight, for instance, the buttocks, hips, elbows, and heels. People who stay in bed for a long time or are unable to move may suffer from PUs, especially the elderly and other vulnerable groups of patients.^[16,17]^ PUs have a significant negative influence on the patients, and they continues to lead to the cost burden on the hospital providers, driven mainly by resources devoted to treating the complications.^[18,19]^ The PUs patients may have longer hospital stays, higher mortality rates during hospitalization, and higher rate of readmission. The special dressings can utilize as the barrier to bacteria, decreasing the pain in the process of healing, absorbing the excess wound fluid, and then generate the suitable conditions for healing and scarring. Several types of dressings have been used in treating PUs and the optimal one remains controversial.

## 5 Conclusion

This study can develop an evidence-based protocol to identify optimal dressings for patients with PUs.

## Author contributions

Fei Zhou designs the protocol. Jun Li reviews the protocol. Shuai Wu performs the data collection. Yan Jin finishes the manuscript. All of the authors approved the submission.

**Conceptualization:** Jun Li.

**Data curation:** Jun Li.

**Funding acquisition:** Fei Zhou.

**Investigation:** Shuai Wu.

**Methodology:** Shuai Wu.

**Writing – original draft:** Yan Jin.

## Abbreviations

Abbreviation: PU = pressure ulcer.
